# Delving into gene-set multiplex networks facilitated by a k-nearest neighbor-based measure of similarity

**DOI:** 10.1016/j.csbj.2023.09.042

**Published:** 2023-10-11

**Authors:** Cheng Zheng, Man Wang, Ryo Yamada, Daigo Okada

**Affiliations:** a Center for Genomic Medicine, Graduate School of Medicine, Kyoto University, South Research Bldg. No.1(5F), 53 Shogoinkawahara-cho, Sakyo-ku, Kyoto, 6068507, Kyoto, Japan; b Department of Signal Transduction, Research Institute for Microbial Diseases, Osaka University, 3-1 Yamadaoka, Suita, 5650871, Osaka, Japan

**Keywords:** Gene set, Multiplex network, k-nearest neighbor-based similarity, Multiplex clustering coefficient, Multiplex PageRank centrality, Gene set enrichment analysis, Gene set co-expression

## Abstract

Gene sets are functional units for living cells. Previously, limited studies investigated the complex relations among gene sets, but documents about their altering patterns across biological conditions still need to be prepared. In this study, we adopted and modified a classical k-nearest neighbor-based association function to detect inter-gene-set similarities. Based on this method, we built multiplex networks of gene sets for the first time; these networks contain layers of gene sets corresponding to different populations of cells. The context-based multiplex networks can capture meaningful biological variation and have considerable differences from knowledge-based networks of gene sets built on Jaccard similarity, as demonstrated in this study. Furthermore, at the scale of individual gene sets, the structural coefficients of gene sets (multiplex PageRank centrality, clustering coefficient, and participation coefficient) disclose the diversity of gene sets from the perspective of structural properties and make it easier to identify unique gene sets. In gene set enrichment analysis (GSEA), each gene set is treated independently, and its contextual and relational attributes are ignored. The structural coefficients of gene sets can supplement GSEA with information about the overall picture of gene sets, promoting the constructive reorganization of the enriched terms and helping researchers better prioritize and select gene sets.

## Highlights

• The KNN-based association function can optimally detect inter-gene-set similarities.
• The multiplex network model can capture gene sets' meaningful biological variation.
• Structural coefficients reflect the multifaceted characteristics of gene sets.
• GSEA can reorganize its terms by integrating gene sets' structural coefficients.

## Graphical abstract

**Figure:**
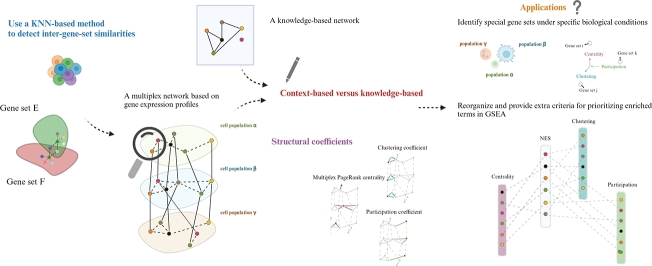

## 1 Introduction

A gene set, by definition, is a set of gene symbols or other equivalent strings with a specific identifier that usually summarizes its essential information [1]. For example, a gene set called “GSE9006_HEALTHY_VS_TYPE_1_DIABETES_PBMC_AT_DX_DN” from the IMMUNESIGDB database includes 198 gene symbols (e.g., CYP4F3, BASP1, and VNN3). Its naming came from case-control microarray research that found these 198 genes down-regulated in peripheral blood mononuclear cells (PBMCs) of healthy subjects compared to newly diagnosed type I diabetes patients [2], [3]. The genes in a gene set may have a mixture of discovery sources, such as biological discovery or computational inference [1], and creation of the set may rely on curators who browse online publications about genes and manually select and annotate genes to form a gene set based on their domain knowledge [4]. Nowadays, gene-set databases are expanding rapidly, and it becomes more and more important to understand the relationship among gene sets that could empower us to identify vital gene sets/pathways or mitigate their redundancy [5], [6], [7].

Many existing models measure the similarity of two gene sets either via memberships or annotation knowledge of their constituent genes (e.g., Jaccard coefficients [8] and kappa statistics [9]) or through gene expression profiles by computing a multivariate association (e.g., analysis of gene co-expression/correlation structures [10] and multivariate associations of gene expression vectors [11]). However, the knowledge-based similarity may differ substantially from the multivariate association inferred from the gene expression because the knowledge is static and ignores the biological context of studies and the complex interactions among genes. While multivariate associations are context-based methods [12], they usually have specific presumptions and significantly higher computational costs for large-scale experiments. Thus, a thorough investigation of multivariate association/similarity measures regarding their practical performance and capability of being applied to gene-set studies is warranted.

In addition, we are interested in the intrinsic features of gene sets in their interacting communities under specific biological conditions. A multiplex network is a mathematical framework that aligns with our purpose [13], [14]. Gene sets can be nodes in the layers of the multiplex network, and the weights of their incident edges can be scalar similarities defined as above. The structural coefficients of a multiplex network integrate the data from all the layers, offering clues to identify characteristic gene sets in the network [15], [16], [17]. Additionally, the findings of network properties of gene sets may help improve the interpretability of gene set enrichment analysis (GSEA) [8], [18]. GSEA and its variants often emphasize the gene-level statistics or effect size/significance of enrichment [19], [20]. The contextual and relational attributes of gene sets are often omitted in GSEA [8], [21]. By delving into the gene set communities, we may have more opportunities to understand the overall picture of gene sets and have extra criteria to select enriched terms in GSEA.

## 2 Methods

### 2.1 General workflow

**Fig. 1:**
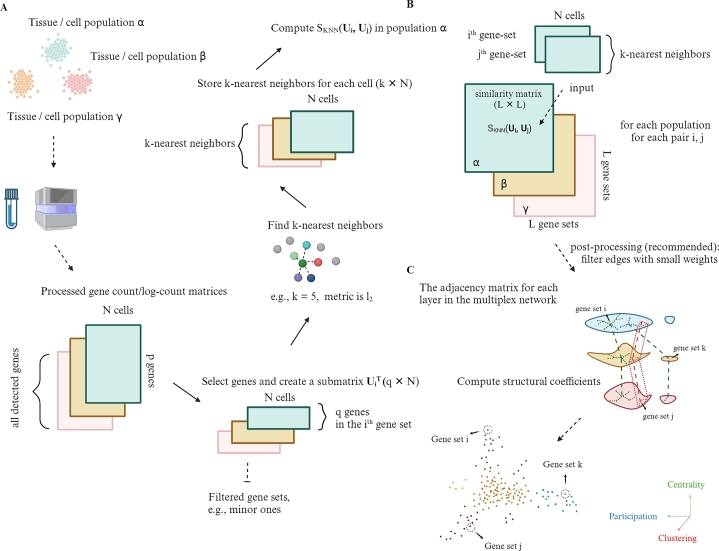
Essential steps in building a multiplex network of gene sets based on $S_{\text{KNN}}$. (A) Cells from three populations *α*,*β*,*γ* are collected and sequenced for their transcripts subjected to further preprocessing. For the simplicity of illustration, we assume that the number of samples in the gene expression matrix of each layer is *N*, and all matrices have the same *p* detected genes. These constraints are not mandatory in practice. For any gene set *i* in a cell population, selecting *q* genes (rows) in the gene set among the *p* genes of the processed gene expression matrix produces a submatrix that determines the distribution of cells in that feature space. Gene sets with too few or too many overlapped genes or that display excessively sparse expression values in the submatrix are excluded. Different layers may have slightly different collections of gene sets that pass the filtration. We take their intersections to make the filtering collections the same. An implemented KNN algorithm with a chosen *k* and a metric will take the *N* × *q* submatrix as the input and return a *N* × *k* index matrix that stores the indices of the k-nearest neighbors for each cell. (B) $S_{\text{KNN}}$ uses a pair of index matrices corresponding to two gene sets to compute the inter-gene-set similarity (section 2.4). By iterating the computation for all pairs of gene sets in all cell populations, three *L* × *L* gene-set similarity matrices are obtained. (C) It is optional but recommended to set a threshold to truncate entries with low values in the similarity matrices to reduce the effects of random noise. The processed similarity matrices can be treated as weighted adjacency matrices for layers in a multiplex network. An example of the downstream analysis of the multiplex network is computing the structural coefficients of gene sets (section 2.7).

A multiplex network consists of a list of regular (monoplex) networks called layers. The layers share the same group of constituent nodes, and the inter-layer relation is simple because a node only connects with its identical node in other layers (for formal definitions, see section 2.6). A real-life example of a multiplex network can be a multiplex social network for a group of people. The same node represents the same person in networks corresponding to different life scenarios, including friendship, hobbies, colleagues, and social media followers. Fig. 1 shows the essential steps in building a multiplex network of gene sets. The analysts can choose and fetch the target collection of gene sets from a database, for example, gene ontology gene sets from org.Hs.eg.db. It also depends on the analysts to determine the data source for computing similarities in each layer of the multiplex network that establishes the biological context for measurement in that layer, which in our experiment is a cell population of a scRNA-seq dataset (section 2.2). Thus, the number of different cell populations equals the number of layers (Fig. 1.A). Other types of samples may work as well.

The Jaccard coefficient is a popular semantic or knowledge-based similarity measure for gene sets. We can modify it to be context-based by adding weights to genes according to their average expression levels in a cell population. Two gene sets are likely dissimilar if their common genes have no expression whereas their private genes dominate, and vice versa. However, the modified Jaccard coefficient cannot capture the total effect of gene expressions on similarities among gene sets because the interactions among genes are crucial and complicated. This is where multivariate association functions may come into play. We designed simulation and running time comparison studies to compare the performance of classical multivariate association functions (for more details, see sections 2.3-2.5). In particular, we focused on a k-nearest-neighbor (KNN)-based association function [12], [22]. Readers can also check the comprehensive review by Josse and Holmes [12].

Once we are using a similarity measure ($S_{\text{KNN}}$ in section 2.4) to compute a list of similarity matrices corresponding to multiple biological conditions, we can filter edges with lower-than-threshold weights and instantiate a multiplex network of gene sets and study its structural properties (section 2.6, Fig. 1.B, C). At a high level, the layer-wise relations signify the connections between biological contexts. For illustration purposes, we select cell populations (subtypes of immune cells) that are well-known to researchers. Other than layers, the distributions of subcollections of gene sets, for example, multiplex communities or families of gene ontology gene sets, can also give insights into the behaviors of a group of gene sets of interest. For example, GO:0070161 (anchoring junction) and GO:0005681 (spliceosomal complex) are two parent gene sets in the level-3 gene ontology-cellular component (GO-CC) gene sets. By observing the distribution of their families, including descendant gene sets, we can get acquainted with all the versions of connections between these two families in both the knowledge-based and context-based networks (section 2.7). To quantify the difference in inter-family connections between the Jaccard network and multiplex networks, we introduce two metrics called within-family and between-family fold changes (section 2.7).

At the scale of individual gene sets, the structural coefficients are multifaceted relational attributes of gene sets about their connectivity across layers, which include a multiplex PageRank centrality, a multiplex clustering coefficient, and a multiplex participation coefficient (Fig. 1.C, section 2.7). The multiplex PageRank centrality and clustering coefficients are generalized from their counterparts in the monoplex network by rewarding “consistent” players with high centrality or the clustering tendency in their neighborhoods across layers. The participation coefficient evaluates the uniformity or bias of connections of a gene set over the layers in a multiplex network. Those heterogeneous features reveal the diversity of gene sets from the structural perspective and are helpful in GSEA applications. Gene sets are independently analyzed in GSEA to estimate their member genes' deviation from a random distribution on a target gene list. Researchers frequently use a singular metric of the effect size, for example, a normalized enrichment score (NES), to determine the priority of gene sets for interpretation. We explored the potential of using the structural coefficients to expand the current scope of GSEA by reorganizing the enriched terms and providing more clues on prioritizing gene sets for better exposing terms deserving attention (section 2.8).

### 2.2 Single-cell RNA-seq datasets and gene sets

The pre-processed single-cell RNA-seq datasets used in this study include pbmc3k.final fetched from SeuratData, which consists of 2638 immune cells collected from the peripheral blood of a healthy human donor [23], and E-MTAB-11536 (EMBL-EBI), which involves 329,762 immune-related human cells from 16 tissues of 12 deceased donors [24]. The pre-processing included quality control, gene count normalization, log transformation, variable gene detection, feature scaling, and data integration when applicable.

The collections of GO-CC and GO biological process (GO-BP) gene sets were accessed through org.Hs.eg.db (version 3.14.0) in gseGO [25], [26], [27], [28]. Their IDs, descriptions, and symbols of constituent genes were formatted and stored in JSON files for further use [29]. ImmuneSigDB (v2022.1/2023.1) is a collection of gene sets stemming from 389 published immunology studies. Researchers manually design comparison experiments for differential gene expression analyses. Upregulated or downregulated genes are packaged as gene sets [3]. They can be downloaded from the Molecular Signatures Database published by the Broad Institute [30].

### 2.3 Notation: similarity matrix of gene sets

Constructing a distance matrix (metric or non-metric) or a similarity matrix can provide insights into the relationship among observations not embedded in a common vector space [31]. For example, let $T=\{U_{1},\cdots ,U_{n}\}$ be a collection of *n* gene sets, and given a similarity function ρ:T×T→R, the n×n matrix with its *ij*-th entry as $\rho (U_{i},U_{j})$ is called a similarity matrix. We note that the similarity functions do not have a common definition, and association functions or the inverse of distance/dissimilar functions can work as similarity measures. The Jaccard coefficient is a popular similarity function for gene sets based on their semantic relationships. In contrast, given gene expression profiles of gene sets (e.g., gene count matrices restricted to the genes in each gene set), we can deploy numerical models to calculate the similarity.

### 2.4 Classical methods for measuring similarity/multivariate association

*Jaccard coefficient*  The Jaccard coefficient is defined as

$$\mathrm{JC}(U,V)=\frac{\left|U\cap V\right|}{\left|U\cup V\right|},$$

where U,V are two gene sets and |⋅| refers to the number of elements in a finite set [32]. To account for the effects of gene expression, we propose a modified Jaccard coefficient by weighing genes according to their average expression levels in a scRNA-seq dataset. If the mean expression level of gene *k* is $\omega_{k}$, then the modified Jaccard coefficient is

$$JC_{\mathrm{mod}}(U,V)=\left\{ \begin{array}{cc} \frac{\sum_{k\in U\cap V}\omega_{k}}{\sum_{k^{\prime }\in U\cup V}\omega_{k^{\prime }}} & \;\text{if}\;\sum_{k^{\prime }\in U\cup V}\omega_{k^{\prime }}\neq 0\\ 0 & \;\text{otherwise} \end{array}\right.$$

The modified RV coefficient, distance correlation coefficient, and Mantel coefficient have closed-form definitions and related tools in Python (Table 1). For the KNN-based association, we gave its definition and used sklearn.neighbors to implement it in Python.

**Table 1:** Functions to measure similarity/multivariate association.

Name	Empirical Formula: f(U,V)	Input→Output	Python Package
**JC**	$\frac{\left|U\cap V\right|}{\left|U\cup V\right|}$	T×T→[0,1]	–
$JC_{\mathrm{mod}}$	$\frac{\sum_{k\in U\cap V}\omega_{k}}{\sum_{k^{\prime }\in U\cup V}\omega_{k^{\prime }}}\;\text{if}\;\sum_{k^{\prime }\in U\cup V}\omega_{k^{\prime }}\neq 0,\;\text{otherwise}\;0$	T×T→[0,1]	–
$RV_{\mathrm{mod}}$	$\frac{\mathrm{tr}((UU^{T}-\mathrm{diag}(UU^{T}))(VV^{T}-\mathrm{diag}(VV^{T})))}{\sqrt{\mathrm{tr}{\left(UU^{T}-\mathrm{diag}(UU^{T})\right)}^{2}\mathrm{tr}{\left(VV^{T}-\mathrm{diag}(VV^{T})\right)}^{2}}}$	$R^{N\times p}\times R^{N\times q}\to [-1,1]$	hoggorm [37]
**dCOR**	$\frac{\mathrm{dCov}(U,V)}{\sqrt{\mathrm{dCov}(U,U)\mathrm{dCov}(V,V)}}\;\text{if denominator}>0\;\text{otherwise}\;0$	$R^{N\times p}\times R^{N\times q}\to [0,1]$	dcor [38]
$⁎⁎r_{M}$	$\frac{\sum_{i,j=1}^{N}(X_{\mathrm{ij}}-\bar{X})(Y_{\mathrm{ij}}-\bar{Y})}{\sqrt{\mathrm{var}(X)\mathrm{var}(Y)}}=\frac{1}{d-1}\sum_{i,j=1}^{N}\frac{X_{\mathrm{ij}}-\bar{X}}{s_{X}}\frac{Y_{\mathrm{ij}}-\bar{Y}}{s_{Y}}$	$R^{N\times N}\times R^{N\times N}\to [-1,1]$	skbio [39]

$\mathrm{diag}(UU^{T})$ is an N×N matrix that only contains the diagonal elements of $UU^{T}$, similarly for $\mathrm{diag}(VV^{T})$.

* Strictly speaking, a map containing the gene expression level of each gene is an additional input for the modified Jaccard function.

** d=N(N−1), $\bar{X}=\frac{\sum_{i,j=1}^{N}X_{\mathrm{ij}}}{d}$, $s_{X}=\sum_{i,j=1}^{N}\frac{{\left(X_{\mathrm{ij}}-\bar{X}\right)}^{2}}{d}$, $\mathrm{var}(X)=d⁎s_{X}$; similarly for **Y**.

*Modified RV coefficient*  Suppose that gene sets *U* and *V* include *p* and *q* detected genes, and gene expression submatrices $U\in R^{N\times p}$ and $V\in R^{N\times q}$ are column-centered, where *N* is the number of cells in the dataset. Inputs **U** and **V** to the **RV** function return an empirical coefficient for their multivariate linear association. The modified RV coefficient ($RV_{\mathrm{mod}}$ in Table 1) was devised to eliminate the dependency on sample size by the original RV coefficient [12], [33].

*Distance correlation coefficient*  With similar notation, the empirical distance covariance (**dCov** in Table 1) for $U\in R^{N\times p}$ and $V\in R^{N\times q}$ is defined as

$$\mathrm{dCov}(U,V)=\frac{1}{N^{2}}\sum_{i,j=1}^{N}X_{\mathrm{ij}}Y_{\mathrm{ij}}$$

where $X_{\mathrm{ij}}=|U_{i.}-U_{j.}{|}_{p}^{\alpha }-\frac{1}{N}\sum_{j=1}^{N}|U_{i.}-U_{j.}{|}_{p}^{\alpha }-\frac{1}{N}\sum_{i=1}^{N}|U_{i.}-U_{j.}{|}_{p}^{\alpha }+\frac{1}{N^{2}}\sum_{i,j=1}^{N}|U_{i.}-U_{j.}{|}_{p}^{\alpha }$; $U_{i.}$ indicates the *i*-th row (sample) of **U**; and $|\cdot {|}_{p}^{\alpha }$ is an Euclidean norm with exponent *α* in $R^{p}$. $Y_{\mathrm{ij}}$ is defined similarly [34]. Then, the empirical distance correlation is defined as

$$\mathrm{dCor}(U,V)=\left\{ \begin{array}{cc} \frac{\mathrm{dCov}(U,V)}{\sqrt{\mathrm{dCov}(U,U)\mathrm{dCov}(V,V)}} & \text{if}\;\mathrm{dCov}(U,U)\mathrm{dCov}(V,V)>0\\ 0 & \text{otherwise} \end{array}\right.$$

Unlike $RV_{\mathrm{mod}}$, **dCov** can detect non-linear associations [12].

*Mantel coefficient*  The inputs for the Mantel function ($r_{M}$ in Table 1) should be two Euclidean distance matrices defined as $X_{\mathrm{ij}}=||U_{i.}-U_{j.}|{|}_{2}$ and $Y_{\mathrm{ij}}=||V_{i.}-V_{j.}|{|}_{2}$, where $U_{i.}$ indicates the *i*-th row vector of **U**. $U_{j.},V_{i.},V_{j.}$ are defined similarly. The function $r_{M}$ can detect non-linear multivariate associations [12], [35].

*KNN-based association*  Given a submatrix **U** and a metric (e.g., L1, L2, or cosine distance), a complete graph and its KNN subgraph can be built where in the KNN graph, each node represents a sample and *k* edges connect between the node and its KNNs (excluding the self-loop). We deployed sklearn.neighbors.NearestNeighbors to find and store the k-nearest neighborhoods for each sample (Fig. 1.A) using the “auto” algorithm and $\sqrt{N}$, with “L2” (Euclidean distance) as the default *k* and metric [36].

Suppose that the KNN graphs for two gene sets have been determined as described above. Friedman and Rafsky proposed a KNN-based coefficient denoted as Γ to measure the cumulative overlaps of the samples' neighborhoods, which in turn reflects the overall resemblance of the multivariate features [22].

$$\Gamma_{U,V}=\frac{1}{2}\sum_{i=1}^{N}\sum_{j=1}^{N}A_{ij}^{\left[U\right]}A_{ij}^{\left[V\right]}=\frac{1}{2}\sum_{i=1}^{N}|{\text{K}}_{U}(i)⋂{\text{K}}_{V}(i)|$$

where $A^{\left[U\right]},A^{\left[V\right]}$ are the adjacency matrices for the KNN graphs built on **U** and **V** [22]; and ${\text{K}}_{U}(i),{\text{K}}_{V}(i)$ are the sets of KNNs of sample *i* (excluding itself) in U,V. We normalized $\Gamma_{U,V}$ by the possible maximum counts of overlapping neighbors to derive $S_{\text{KNN}}$ as follows (Fig. 1.B):

$$S_{\text{KNN}}(U,V)=\frac{2\Gamma_{U,V}}{kN}\in [0,1]$$

### 2.5 Simulation and comparison studies

**Fig. 2:**
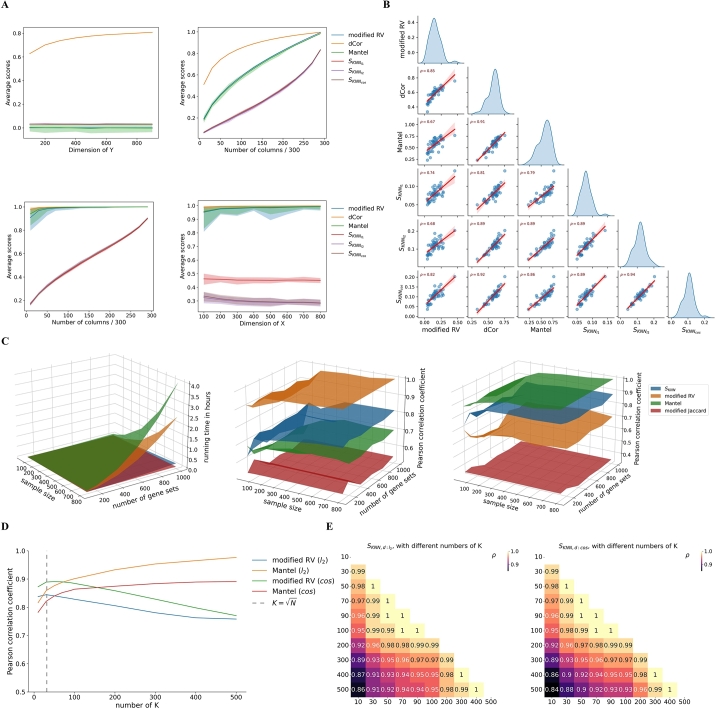
Results of the simulation and comparison studies. (A) Upper left: Most similarity measures exhibit close-to-zero scores for a pair of independent datasets, except for **dCOR** (exponent = 1). Upper right, lower left: $S_{\text{KNN}}$ captures the similarity gradient between a dataset and its subset with increasingly overlapped features. Lower right: The similarity corresponding to a non-linear function remains relatively stable with inputs of various dimensions. The widths of colored bands represent the range of scores in 50 repeated experiments. (B) Outcomes by different similarity measures for real gene sets. (C) The running time of **RV**_**mod**_ and **r**_**M**_ grows much faster than that of $S_{\text{KNN}}$ and **JC**_**mod**_ as the sample size or number of gene sets increases (left). The measurement by **JC**_**mod**_ does not correlate well with the outcomes of **RV**_**mod**_ (middle) or **r**_**M**_ (right), though it has the shortest running time. (D, E) The impact of hyperparameters *K* and metrics on the performance of $S_{\text{KNN}}$.

In the first simulation experiment, two multivariate Gaussian random vectors $X∼N(0,I_{p}),Y∼N(0,I_{q})$ in two feature spaces are independent, that is, X⊥Y, and 1000 observations are drawn from the joint distribution of X,Y. We fix *p* to be 300 and assign *q* to be 100,200,⋯,800 (Fig. 2.A, upper left). In the second experiment, $X∼N(0,I_{p})$, and $Y_{1}=X_{1},\cdots ,Y_{q}=X_{q},q\leq p=300$; that is, realizations of **Y** are subvectors of realizations of **X**. The overlap between X,Y gradually increases as q=10,30,⋯,270 (Fig. 2.A, upper right). In the third experiment, **X** and **Y** maintain the same relationship, but now **X** is a random vector of a Gaussian mixture distribution such that $X|Z=i∼N(A\mu_{i},AA^{T}),i=1,2,3$, where $\mu_{i}\in R^{p}$ is a random mean vector and $A\in R^{p\times p}$ is randomly generated to increase the anisotropy of data in each subpopulation; **Z** specifies the component memberships of observations. Observed data are equally drawn from three subpopulations (Fig. 2.A, lower left). In the fourth experiment, **X** is the same random vector as in the third experiment, and $Y=f(X)=\alpha X+\beta X^{\circ 3}$, where α,β∈R are scalar constants and ∘ indicates the element-wise Hadamard power. *p* is assigned to be 100,200,⋯,800 (Fig. 2.A, lower right). Finally, we measure the similarities of 50 random pairs of ImmuneSigDB gene sets. The cells for computing similarities are 1000 sampled classical monocytes in the blood of the E-MTAB-11536 dataset (Fig. 2.B). $RV_{\mathrm{mod}},\mathrm{dCOR}\;(\text{exponent}=1),r_{M}$, and $S_{\text{KNN}}$ with metrics $l_{1},l_{2},cos$ and $k=31\approx \sqrt{1000}$ are deployed to measure the associations as described in previous sections. The experiment is repeated 50 times for each given *p* and *q*.

Meanwhile, we perform a running time comparison of $RV_{\mathrm{mod}},r_{M},JC_{\mathrm{mod}}$, and $S_{\text{KNN}}$ with the default *k* and metric that all support parallel computations. The sample size is 100,200,⋯,800, and the number of gene sets is 200,400,⋯,1000. The cells are sampled from classical monocytes in the blood of the E-MTAB-11536 dataset, and gene sets are sampled from ImmuneSigDB (v2022.1). We record the similarity matrices computed by the tested methods with different sample sizes or numbers of gene sets and their computing time. The Pearson correlation of two flattened upper triangles (above the main diagonals) of similarity matrices is used to quantify the linear association of two similarity measurements [40]. To justify the impact of sample size, given a fixed collection of gene sets, we treat the measurements of $RV_{\mathrm{mod}}$ or $r_{M}$ under maximal sample size as the reference and compute the Pearson correlations between them and other similarity measurements (Fig. 2.C). The machine used in this part is a desktop workstation (HP Z640) with 10 CPUs (1.70 GHz Xeon) and 64 GB of RAM. Moreover, to explore the impact of hyperparameters (*k* and metrics) on the performance of $S_{\text{KNN}}$, we fix the sample size to be 1000 and randomly choose 100 ImmuneSigDB gene sets, with either one of the metrics ($l_{2}$ or *cos*) and different *k* values (Fig. 2.D, E).

### 2.6 Representation of a multiplex network for gene sets

A multiplex network is a special subtype of a multilayer network. The latter is a triple $M=(\text{Y},\overset{\to }{G},G)$, where Y={α:α∈{1,⋯,M}} is an index set for the *M* layers; $\overset{\to }{G}=(G_{1},\cdots ,G_{\alpha },\cdots ,G_{M})$ is an ordered list of networks (layers); $G_{\alpha }=(V_{\alpha },E_{\alpha })$ is an individual network where $V_{\alpha }$ is the set of nodes and $E_{\alpha }$ contains edges that can be either directed or undirected and weighted or unweighted; and G is a list of bipartite networks such as $G_{\alpha ,\beta }=(V_{\alpha },V_{\beta },E_{\alpha ,\beta })$, where α,β∈Y,α≠β. In other words, G depicts the pairwise connections between different networks in $\overset{\to }{G}$ [13].

When the nodes in $V_{\alpha }$ remain the same for α∈Y (so-called replica nodes) and edges between any pair of distinct layers connect exclusively to the identical replica nodes, the multilayer network M is called a multiplex network. Specifically, if M is an unweighted multiplex network, then the |V|M×|V|M matrix A, called the supra-adjacency matrix, can describe its structure compactly as

$$A=\left(\begin{array}{cccc} A^{\left[1\right]} & I & \cdots & I\\ I & A^{\left[2\right]} & \cdots & I\\ \vdots & \vdots & \ddots & \vdots \\ I & I & \cdots & A^{\left[M\right]} \end{array}\right)$$

where $A^{\left[\alpha \right]},\alpha \in Y$ is the adjacency matrix for $G_{\alpha }$ [13].

A similarity matrix of gene sets can be treated as an adjacency matrix for a layer if its entries are non-negative, for example, $\rho =S_{\text{KNN}}$. Furthermore, a preset positive threshold can filter edges with low weights, reducing the unwanted effects of random noise. Given a list of adjacency matrices for gene sets with the edges exclusively linked to identical gene sets, we can implement a multiplex network that satisfies the formal definition above.

### 2.7 Exploration of structural properties of a gene-set multiplex network

*High-level properties of a gene-set multiplex network*  We first explore the relations among the layers and subcollections of gene sets at a high level. One way to detect the inter-layer connections is to first cluster nodes in each layer of the multiplex network [41], and then use the normalized mutual information (NMI, sklearn.metrics.normalized_mutual_info_score) to quantify the resemblance of cluster structures, which in turn reflects their layer-wise similarities [15]. The seaborn.clustermap displays the layer-wise similarities with a dendrogram showing the hierarchical clusters of layers under the default setting [42], [43]. A multiplex community is a collection of gene sets with connections across multiple layers. The Leiden algorithm can be generalized for community detection in a multiplex network. The quality function for a multiplex network is defined as a weighted sum of quality in each layer as $Q(M,P)=\sum_{k}w_{k}Q(G_{k},P)$, where *P* is a partition of nodes that is the same for all layers and $w_{k}$ is a weight for the *k*-th layer that can be uniform. We deploy leidenalg.ModularityVertexPartition in Python for this task, and the optimization process is the same as that in a monoplex network [41]. Another important example of a subcollection of gene sets is a family of gene ontology gene sets, including a parent gene set and its descendants. For example, GO-CC (cellular components) gene sets have a tree-like organizational structure that a directed acyclic graph can represent. The difference is that some gene sets may have more than one parent. The connections between two families of gene sets may change drastically in layers of the multiplex network compared to the knowledge-based Jaccard network. To quantify this difference, we propose a fold change of similarities as follows:

$$\text{FC}(F,H)=\frac{\frac{1}{M|F||H|}\sum_{i=1}^{M}\sum_{U\in F,V\in H}S_{\text{KNN}}(U_{i},V_{i})}{\frac{1}{\left|F||H\right|}\sum_{U\in F,V\in H}\mathrm{JC}(U,V)}=\frac{\sum_{i=1}^{M}\sum_{U\in F,V\in H}S_{\text{KNN}}(U_{i},V_{i})}{M\sum_{U\in F,V\in H}\mathrm{JC}(U,V)}$$

where *M* is the number of layers in M; and $U_{i},V_{i}$ are gene expression matrices corresponding to the gene sets U,V in the ${\text{i}}^{\text{th}}$ layer. Alternatively speaking, **FC** is the ratio between the average score of $S_{\text{KNN}}$ for all pairs of gene sets U,V in two families F,H across all layers of M and their average **JC** score in the Jaccard network. When F,H are the same family of gene sets, we call it a within-family fold change. If the numerator and denominator are zero, we assign their **FC** as a missing value. If only the denominator is zero, **FC** is conservatively evaluated to be 1. To consider the overall context-over-knowledge difference in the connections between a family of gene sets F and other families, for example, the collections of level-3 GO-CC gene sets and their descendants, we define the between-family fold change as

$$\frac{\sum_{H\neq F,H\in \Omega }\mathrm{FC}(F,H)}{|\Omega |-1}$$

where Ω is a collection of families. The python package goatools is used in this task to search level-3 GO-CC gene sets and their descendants [44].

We use the Uniform Manifold Approximation and Projection for Dimension Reduction (UMAP) and graphical interface to visualize multi-omics networks (Grimon) to visualize the distributions of gene sets. UMAP assumes that the data are distributed uniformly on a manifold, and computationally, it modifies the raw distance/dissimilarity matrix and embeds the newly constructed graph in the low-dimensional space via a force-directed graph layout algorithm [45]. UMAP suits our needs as a fast and straightforward tool without displaying excessive information such as edges. Additionally, we apply Grimon to aggregate and adjust the UMAP coordinates of all the layers in the multiplex network for a unified view [46].

*Structural coefficients of gene sets*  At the scale of individual nodes (gene sets), several structural coefficients have been proposed for a multiplex network with unweighted and undirected layers [15], [16], which can be generalized for multiplex networks with weighted (or directed) layers. The multiplex participation coefficient for node *i* in a multiplex network M with weighted layers can be

$$P_{i}=\frac{M}{M-1}(1-\sum_{\alpha \in \text{Y}}{\left(\frac{k_{i}^{\alpha }}{O_{i}}\right)}^{2})$$

where $k_{i}^{\alpha }=\sum_{j\neq i}A_{ij}^{\left[\alpha \right]}$ is the sum of weights for incident edges of a node *i* at layer *α* and $O_{i}=\sum_{\alpha \in \text{Y}}k_{i}^{\alpha }$. A large $P_{i}$ implies that node *i* has consistent connections across layers. If $P_{i}$ is as low as 0, node *i* has connectivity at a single layer [15].

The (local) clustering coefficient of node *i* quantifies the tendency of nodes in its (open) neighborhood to form connected links (together as triangles) in a network, which is initially proposed for unweighted and undirected graphs as $C_{i}=\frac{\sum_{j\neq i,k\neq i,k\neq j}A_{ij}A_{jk}A_{ki}}{\sum_{j\neq i,k\neq i,k\neq j}A_{ij}A_{ki}}=\frac{\sum_{j\neq i,k\neq i,k\neq j}A_{ij}A_{jk}A_{ki}}{K_{i}(K_{i}-1)}$, where A is an adjacency matrix and $K_{i}=\sum_{j\neq i}A_{ij}$ [47]. For weighted and undirected networks, $A_{ij}$ can be the non-negative normalized weight of edge $E_{ij}$ (i.e., ${\overset{˜}{w}}_{ij}=\frac{w_{ij}}{\max_{ij}w_{ij}}$), and $K_{i}(K_{i}-1)$ becomes ${\left(\sum_{j}{\overset{˜}{w}}_{ij}\right)}^{2}-\sum_{k}{\overset{˜}{w}}_{ki}^{2}$ [48], [49]. In the case of directed and weighted graphs, we may consider a specific pattern of triangles with fixed edge directions (e.g., $C_{i}=\frac{\sum_{j\neq i,k\neq i,k\neq j}{\overset{˜}{w}}_{ij}{\overset{˜}{w}}_{ik}{\overset{˜}{w}}_{jk}}{\sum_{j\neq i,k\neq i,k\neq j}{\overset{˜}{w}}_{ij}{\overset{˜}{w}}_{ik}}$) [50] or all possible triangles that contain node *i* as their vertex (i.e., $C_{i}=\frac{\sum_{j\neq i,k\neq i,k<j}({\overset{˜}{w}}_{ij}+{\overset{˜}{w}}_{ji})({\overset{˜}{w}}_{ik}+{\overset{˜}{w}}_{ki})({\overset{˜}{w}}_{jk}+{\overset{˜}{w}}_{kj})}{2\sum_{j\neq i,k\neq i,k<j}({\overset{˜}{w}}_{ij}+{\overset{˜}{w}}_{ji})({\overset{˜}{w}}_{ik}+{\overset{˜}{w}}_{ki})}$) [51], [52].

An extended version for a multiplex network can be developed by placing the edges of a triangle on two or three different layers, which correspond to coefficients $C_{i,1}$ and $C_{i,2}$ [15], [16]. Without loss of generality, we assume that $G_{\alpha }$ is directed and weighted and has no self-loops for all α∈Y. The equations in the definitions can be transformed into matrix forms to facilitate vectorization and parallel processing. The proof is in the appendix.

$$C_{i,1}=\frac{\sum_{\alpha }\sum_{\alpha^{\prime }\neq \alpha }\sum_{j\neq i,m\neq i,j<m}(A_{ij}^{\left[\alpha \right]}+A_{ji}^{\left[\alpha \right]})(A_{jm}^{\left[\alpha^{\prime }\right]}+A_{mj}^{\left[\alpha^{\prime }\right]})(A_{mi}^{\left[\alpha \right]}+A_{im}^{\left[\alpha \right]})}{2(M-1)\sum_{\alpha }\sum_{j\neq i,m\neq i,j<m}(A_{ij}^{\left[\alpha \right]}+A_{ji}^{\left[\alpha \right]})(A_{mi}^{\left[\alpha \right]}+A_{im}^{\left[\alpha \right]})}=\frac{\sum_{\alpha }\sum_{\alpha^{\prime }\neq \alpha }\mathrm{Diag}{\left((A^{\left[\alpha \right]}+A^{{\left[\alpha \right]}^{T}})(A^{\left[\alpha^{\prime }\right]}+A^{{\left[\alpha^{\prime }\right]}^{T}})(A^{\left[\alpha \right]}+A^{{\left[\alpha \right]}^{T}})\right)}_{ii}}{2(M-1)\sum_{\alpha }{\left({\left((A^{\left[\alpha \right]}+A^{{\left[\alpha \right]}^{T}})1\right)}^{\circ 2}-{\left(A^{\left[\alpha \right]}+A^{{\left[\alpha \right]}^{T}}\right)}^{\circ 2}1\right)}_{i}}\;C_{i,2}=\frac{\sum_{\alpha }\sum_{\alpha^{\prime }\neq \alpha }\sum_{\alpha^{\prime \prime }\neq \alpha ,\alpha^{\prime }}\sum_{j\neq i,m\neq i,j\neq m}(A_{ij}^{\left[\alpha \right]}+A_{ji}^{\left[\alpha \right]})(A_{jm}^{\left[\alpha^{\prime \prime }\right]}+A_{mj}^{\left[\alpha^{\prime \prime }\right]})(A_{mi}^{\left[\alpha^{\prime }\right]}+A_{im}^{\left[\alpha^{\prime }\right]})}{2(M-2)\sum_{\alpha }\sum_{\alpha^{\prime }\neq \alpha }\sum_{j\neq i,m\neq i,j\neq m}(A_{ij}^{\left[\alpha \right]}+A_{ji}^{\left[\alpha \right]})(A_{mi}^{\left[\alpha^{\prime }\right]}+A_{im}^{\left[\alpha^{\prime }\right]})}=\frac{\sum_{\alpha }\sum_{\alpha^{\prime }\neq \alpha }\sum_{\alpha^{\prime \prime }\neq \alpha ,\alpha^{\prime }}\mathrm{Diag}{\left((A^{\left[\alpha \right]}+A^{{\left[\alpha \right]}^{T}})(A^{\left[\alpha^{\prime \prime }\right]}+A^{{\left[\alpha^{\prime \prime }\right]}^{T}})(A^{\left[\alpha^{\prime }\right]}+A^{{\left[\alpha^{\prime }\right]}^{T}})\right)}_{ii}}{2(M-2)\sum_{\alpha }\sum_{\alpha^{\prime }\neq \alpha }{\left(((A^{\left[\alpha \right]}+A^{{\left[\alpha \right]}^{T}})1)⊙((A^{\left[\alpha^{\prime }\right]}+A^{{\left[\alpha^{\prime }\right]}^{T}})1)-((A^{\left[\alpha \right]}+A^{{\left[\alpha \right]}^{T}})⊙(A^{\left[\alpha^{\prime }\right]}+A^{{\left[\alpha^{\prime }\right]}^{T}}))1\right)}_{i}}$$

We refer to a multiplex version of the PageRank coefficient, which reflects the global centrality of a node in a multiplex network, and implement it in Python [17]. The algorithm attempts to integrate centrality information from layers iteratively. Suppose that the adjacency matrices $\left\{A^{\left[L_{k}\right]}\right\}$ of layers $\left(L_{1},\cdots ,L_{m}\right)$ in a multiplex network are column-normalized (i.e., $\sum_{i=1}^{N}A_{ij}^{\left[L_{k}\right]}=1$ for all k,j), and the multiplex PageRank centrality for nodes in layers $\left(L_{1},\cdots ,L_{k-1}\right)$ denoted as $X^{k-1}={\left[x_{1}^{k-1},\cdots ,x_{N}^{k-1}\right]}^{T}$ has already been determined. Then, the multiplex PageRank centrality for node *i* in layers $\left(L_{1},\cdots ,L_{k}\right)$ is

$$x_{i}^{k}=\alpha_{L_{k}}\sum_{j=1}^{N}{\left(x_{i}^{k-1}\right)}^{\beta }A_{ij}^{\left[L_{k}\right]}\frac{x_{j}^{k}}{g_{j}^{k}}+(1-\alpha_{L_{k}})\frac{{\left(x_{i}^{k-1}\right)}^{\gamma }}{\sum_{r=1}^{N}{\left(x_{r}^{k-1}\right)}^{\gamma }}$$

where k=2,⋯,m; $g_{j}^{k}=\sum_{r=1}^{N}A_{rj}^{\left[L_{k}\right]}{\left(x_{r}^{k-1}\right)}^{\beta }+\delta (0,\sum_{r=1}^{N}A_{rj}^{\left[L_{k}\right]}{\left(x_{r}^{k-1}\right)}^{\beta })$; *δ* is the Kronecker delta function; and $\alpha_{L_{k}},\gamma ,\beta$ are scalar hyperparameters (defaults are respectively 0.85, 1, and 1 in our experiments) [17]. The equation can be expressed more concisely as

$$X^{k}=\alpha_{L_{k}}\overset{ˆ}{M}X^{k}+(1-\alpha_{L_{k}})\frac{{\left(X^{k-1}\right)}^{\circ \gamma }}{1^{T}{\left(X^{k-1}\right)}^{\circ \gamma }}$$

where $M=DA^{\left[L_{k}\right]}$, $\overset{ˆ}{M}$ is its column-normalized form (entries in a column remain zeros if divided by a zero column sum), and **D** is a diagonal matrix such that $D_{ii}={\left(x_{i}^{k-1}\right)}^{\beta }$. The base case is when k=1 and ${\left[x_{1}^{1},\cdots ,x_{N}^{1}\right]}^{T}$ is defined to be the same as the PageRank centrality of a monoplex network $L_{1}$ [53]. The numeric computation is through iterative procedures, and the final converged multiplex PageRank centrality for node *i* is $x_{i}=x_{i}^{m}$ [17]. We use the ratio of raw centrality to a uniform mass $\frac{1}{\text{number of gene sets}}$ to represent the centrality of nodes in this study.

### 2.8 Applications in gene set enrichment analysis

GSEA is a popular tool for interpreting the functional meaning of a target gene list. Given an ordered list of *n* weighted genes $\Omega =\{g_{1},\cdots ,g_{n}\}$, for example, differentially expressed genes ranked by a metric for differences or adjusted p-values in hypothesis tests, the enrichment score (**ES**) for a gene set *U* is defined as follows:

$${\text{S}}_{\text{hit}}(U,i)=\sum_{\begin{array}{c} g_{k}\in U\\ k\leq i \end{array}}\frac{|\rho_{k}{|}^{\alpha }}{\sum_{g_{k}\in U}|\rho_{k}{|}^{\alpha }}\;{\text{S}}_{\text{miss}}(U,i)=\sum_{\begin{array}{c} g_{k}\notin U\\ k\leq i \end{array}}\frac{1}{n-|U|}\;p=\underset{i}{\mathrm{arg max}}|{\text{S}}_{\text{hit}}(U,i)-{\text{S}}_{\text{miss}}(U,i)|\text{ES}(U)={\text{S}}_{\text{hit}}(U,p)-{\text{S}}_{\text{miss}}(U,p)$$

where p,i are positions in the ordered list of genes, $\rho_{k}$ is the weight for the gene $g_{k}$, and *α* is a scalar hyperparameter [18]. By permutations, a null distribution can be built. The proposal of the NES aims to reduce the effect of different sizes of gene sets in multiple-hypothesis testing by rescaling the enrichment score [18]. For a gene set *U* with a highly positive or negative NES, the genes in *U* are unlikely to be randomly distributed in Ω, which implies the existence of their underlying connection. We use clusterProfiler (version 3.17) in R to conduct GSEA [26].

Practitioners often deal with many enriched gene sets in an experiment. A typical routine to determine the priority of enriched terms is to rank them by their (normalized) enrichment scores, gene overlap ratio (GeneRatio), or adjusted p-values, and only the gene sets with top ranks are emphasized. Those metrics mainly consider the effect size or statistical significance of the distributional deviation of genes. Some researchers may even pick their terms of interest, regardless of the ranks of those gene sets. However, a neurological term enriched in a study about gliomas may have a fundamentally different meaning if it were enriched in a rheumatology study. GSEA treats each gene set independently, and the interpretation of enriched terms does not explicitly consider contextual and relational attributes of gene sets, which the structural coefficients can supplement.

We use the multiplex clustering coefficients (C1 and C2) as indicators to find local clusters of gene sets with significant similarities by searching the “consistent” neighbors of gene sets with high-level clustering coefficients, for example, sharing the neighborhood over the layers ($S_{\text{KNN}}>0.2$). Then, rather than relying on the knowledge of gene sets' hierarchical organizations [8], [21], local representatives of the groups are selected if they have maximal (local) multiplex PageRank centrality in the groups, which produces context-based representatives of the groups in a data-driven manner. The gene sets other than the representatives in the group are removed from the enriched terms. As many terms with high (global) multiplex centrality are gene sets too ambiguous for interpretation, we can concentrate on more specific terms with lower multiplex centrality. Regarding the multiplex participation coefficient, ${\left(\frac{k_{i}^{\alpha }}{O_{i}}\right)}^{2}\in [0,1]$ (section 2.7) is a scalar value to reflect the biased “participation” of a gene set *i* in a particular layer *α*. By examining gene sets' participation coefficients and per-layer participation, we know the specificity of their connectivities to particular layers. To combine them, we use multiplex clustering coefficients and PageRank centrality to reorganize the raw structures of enriched terms in GSEA to reduce redundancy and skip enriched terms with overly broad functions. Then, we can prioritize gene sets by both NES and multiplex centrality levels. The alternative way is to follow the orders of structural coefficients of gene sets, for example, multiplex participation coefficients, depending on the interest of researchers. We find that the multipartite graph is a valuable tool (networkx.multilayered_graph) to visualize the enriched terms ranked by different standards in a sensible way [54].

## 3 Results and discussion

### 3.1 Computing performance of $S_{\text{KNN}}$

As described in section 2.5, we first conduct a simulation study to test the ability of selected similarity measures to reflect various simulated relations (Fig. 2.A, B). For simulation dataset 1, each data point *i* has the same probability $\frac{K}{N-1}$ of being the KNN of another point *j*, and the neighborhoods in the random matrices X and Y are also independent. Thus, we can estimate the expectation of $S_{\text{KNN}}(X,Y)$ as $E[\frac{2⁎\frac{1}{2}\sum_{i=1}^{N}|{\text{K}}_{X}(i)⋂{\text{K}}_{Y}(i)|}{KN}]=\frac{1}{KN}\sum_{i=1}^{N}E[|{\text{K}}_{X}(i)⋂{\text{K}}_{Y}(i)|]=\frac{1}{KN}\sum_{i=1}^{N}\sum_{j=1}^{N}E[1_{j\in {\text{K}}_{X}(i)⋂{\text{K}}_{Y}(i)}]=\frac{N(N-1){\left(\frac{K}{N-1}\right)}^{2}}{KN}=\frac{K}{N-1}$. In this experiment, $K=31\approx \sqrt{N}$, and we observe the scores of $S_{\text{KNN}}$ fluctuate on its expectation $\frac{K}{N-1}\approx 0.03103$ with a little variance no matter which metric is loaded (Fig. 2.A, upper left). In other words, for two completely random and independent datasets, $S_{\text{KNN}}$ will give a near-zero similarity score in the choice of a reasonable *K* (e.g., $\sqrt{N}$). The difference between simulation datasets 2 and 3 is that dataset 2 has no internal structures while dataset 3 has three separate clusters. $S_{\text{KNN}}$ smoothly captures the gradient of similarity between a data matrix and its submatrix as the number of columns increases. However, other similarity measures might be too sensitive to the global structures of datasets as their similarity scores approach 1 as soon as the submatrix recruits about 50 (over 300) columns of the raw data matrix (Fig. 2.A, upper right and lower left). Given a non-linear function, $S_{\text{KNN}}$ and other similarity measures can assign a non-trivial and consistent score to it regardless of the dimension of the input dataset, though different metrics embedded in $S_{\text{KNN}}$ may lead to divergent scores (Fig. 2.A, lower right). Fig. 2.B shows the correlations between pairs of different similarity measures for 50 ImmuneSig gene sets. $S_{\text{KNN}}$ with default metric $l_{2}$ and $K=\sqrt{N}$ has an intermediate correlation (0.68) with $RV_{\mathrm{mod}}$, but a strong correlation (0.89) with $r_{M}$.

We then perform a running time comparison experiment on $RV_{\mathrm{mod}},r_{M},JC_{\mathrm{mod}}$, and $S_{\text{KNN}}$ with the default metric and *K*. For an execution with 800 samples and 1000 target gene sets, $S_{\text{KNN}}$ outperforms $RV_{\mathrm{mod}}$ and $r_{M}$ remarkably having more than one order of magnitude less time cost (10.4 minutes versus 2.4 and 4.0 hours). $JC_{\mathrm{mod}}$ has an even lower time consumption (Fig. 2.C, left) but at the cost of worse performance. For simplicity of discussion, we assume that all *L* gene sets have the same size as *s*, and the computational complexity of the multiplication of an N×s matrix and its transpose is $O(sN^{2})$. Then, from section 2.4 and Table 1, the time complexities of $RV_{\mathrm{mod}}$ and $r_{M}$ are $O(sN^{2}+N^{3})$ and $O(sN^{2})$, respectively. For computing the L×L similarity matrix, their time complexities are $O[(sN^{2}+N^{3})L^{2}]$ and $O(sN^{2}L^{2})$, respectively. In contrast, the time complexity of computing the *L* index matrices (Fig. 1) is $O(sN^{2}L)$ by the brute algorithm of sklearn.neighbors. Computing $S_{\text{KNN}}$ based on a pair of index matrices (Fig. 1) would require the complexity of O(KN). Thus, the total complexity for computing the similarity matrix is $O(sN^{2}L+KNL^{2})$. The time complexity of $JC_{\mathrm{mod}}$ for computing the similarity matrix is $O(sL^{2})$ given a map of average gene expressions of genes as its input.

Regarding the performance, the measurements by $S_{\text{KNN}},RV_{\mathrm{mod}},r_{M}$ are mutually associated and influenced by the sample size. With the measurement by $RV_{\mathrm{mod}}$ or $r_{M}$ under the largest sample size as a reference (Fig. 2.C, middle right), the correlations between the reference and the measurements by $RV_{\mathrm{mod}}$ or $r_{M}$ under different sample sizes gradually become stable as the sample size increases. Because the cells in this experiment are sampled from a homogenous population, a requirement for a larger sample size for the convergence of measurements is expected if they were sampled from a heterogeneous population. The correlations between the reference and measurements by $S_{\text{KNN}}$ lie between $RV_{\mathrm{mod}}$ and $r_{M}$. For example, compared to $r_{M}$, $S_{\text{KNN}}$ is closer to $RV_{\mathrm{mod}}$ and farther from $r_{M}$. The measurements by $JC_{\mathrm{mod}}$ have poor Pearson correlations with both methods $RV_{\mathrm{mod}}$ and $r_{M}$, which suggests that gene membership and expression levels alone cannot explain the interactions among gene sets.

We further explore the impact of *K* and metrics on the outcomes of $S_{\text{KNN}}$. With an increasing number of *K* ($<\frac{1}{2}N$), $S_{\text{KNN}}$ has the tendency of approaching the non-linear association function $r_{M}$ and deviating from the linear association function $RV_{\mathrm{mod}}$ with both metrics $l_{2}$ and *cos* (Fig. 2.D). $S_{\text{KNN}}$ loaded with the $l_{2}$ metric is significantly closer to $r_{M}$ than when loaded with the *cos* metric (p-value = 0.002 by Wilcoxon signed-rank test). Oppositely, $S_{\text{KNN}}$ loaded with the *cos* metric is significantly closer to $RV_{\mathrm{mod}}$ than when loaded with $l_{2}$ (p-value = 0.002 by Wilcoxon signed-rank test). Thus, the hyperparameters *K* and metrics can adjust the inclination of $S_{\text{KNN}}$ towards a linear or non-linear similarity measure. But in general, a change of hyperparameter *K* does not abruptly switch the effect of measurement: for *K* in the interval around 100, the outcomes of $S_{\text{KNN}}$ correlate strongly (Fig. 2.E).

Similarity measures like $S_{\text{KNN}}$ have some limitations. Unlike the deterministic semantic relations, the quality of gene expression datasets influences the computational results. For example, a scRNA-seq dataset with low coverage depth may have few detected genes for gene sets, resulting in a sparse count matrix that is unreliable in predicting the similarity. Due to the stochastic expression nature of genes, especially regulatory genes, the current scRNA-seq technology may not reliably capture their gene expression levels, which may underestimate their connectivity in the community [55]. In addition, the current exclusion criteria may reject some “true negative” gene sets that are silent under specific contexts.

### 3.2 The multiplex network model can capture the biological variation of gene sets in context

**Fig. 3:**
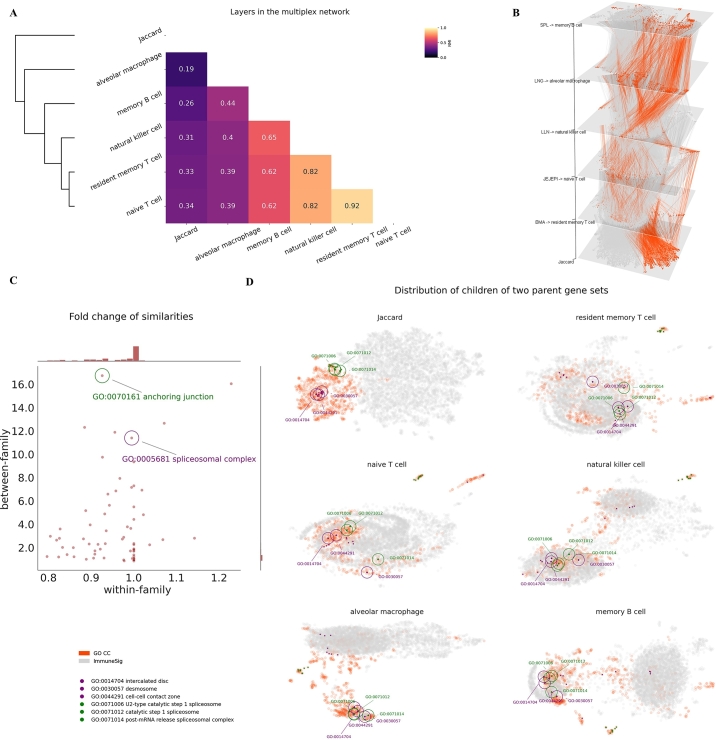
Difference between the knowledge-based Jaccard network and the context-based multiplex network. (A) NMI scores indicate similarity between pairs of layers. The NMI scores and the hierarchical clustering show that the Jaccard layer differs significantly from all other layers in the multiplex network. (B) View of the distributions of GO-CC and ImmuneSig gene sets by Grimon. Each splice corresponds to a plot in D by its label. The coordinates of gene sets in the slices are UMAP coordinates adjusted by Grimon. There is a pronounced change in terms of the interactions between gene sets of GO-CC and ImmuneSig in the Jaccard network and layers in the multiplex network. (C, D) Most level-3 GO-CC gene sets and their descendants show within-family fold changes around 1. In contrast, their between-family fold changes can be much higher. Families GO:0070161 and GO:0005681 are parent gene sets. Their between-family relations change remarkably from the knowledge-based Jaccard layer to layers in the multiplex network, as visualized by the distributions of their family gene sets in the embedded UMAP space. Some of their descendant gene sets (marked by circles) become close in the multiplex network embedding.

We use $S_{\text{KNN}}$ with the default setting to compute similarities among gene sets in GO-CC and ImmuneSig. Five cell populations from human donors (EMBL-EBI) that include memory B cells in spleens, alveolar macrophages in lungs, natural killer cells in lung-draining lymph nodes, naive T cells in jejunum epithelium, and resident memory T cells in bone marrow set up the biological context for the measurements, corresponding to layers in the multiplex network (Fig. 3). We exclude gene sets that are too small or too large, namely, less than 10 or greater than 2000 genes in a gene expression matrix. To mitigate noise in the network, edges whose weights are lower than a positive threshold (e.g., 0.2) are discarded. We add an auxiliary network built on the Jaccard similarity matrix as a knowledge-based reference.

The layer-wise similarities are measured by NMI, and hierarchical clustering is performed (section 2.7). Layers for T cells and natural killer cells are clustered close to the layer for B cells. The layer for macrophages is on another branch of the hierarchical tree (Fig. 3.A). The hierarchical structure of layers in the multiplex network fits the model of hematopoiesis and the classification of peripheral agranular leukocytes in physiological states [56], which demonstrates the efficacy of multiplex networks for capturing a genuine biological variation.

In contrast, the knowledge-based Jaccard network has the least NMI with all layers in the multiplex network. In the UMAP space of the embedded Jaccard layer, GO-CC gene sets barely cover ImmuneSig gene sets. Conversely, these two groups of gene sets often significantly overlap in the UMAP space of embedded layers of the multiplex network (Fig. 3.D). The difference between a knowledge-based and a context-based network is prominent in this example. From a biological standpoint, this is reasonable, as gene sets in ImmuneSig rely on specific molecular machinery to conduct immunological or other elementary functions. Because GO-CC gene sets have a tree-like hierarchical structure, we are interested in how the difference between knowledge-based and context-based networks is decomposed into within-family and between-family portions (section 2.7).

The chosen families of gene sets are level-3 GO-CC gene sets and their descendants. Our finding (Fig. 3.C) shows that the between-family fold changes (median 2, range 0.86-16.7) are remarkably higher than within-family fold changes (median 0.99, range 0.8-1.23). For instance, the family GO:0070161 (anchoring junction) exhibits the highest ratio of these two metrics. It has a within-family fold change of 0.93, implying its within-family relations are, on average, similar to those found in the knowledge-based reference. In contrast, its between-family fold change with other families is 16.74, indicating that their contextual relations are quite different from those in the knowledge-based approach. A specific example is the family GO:0005681 (spliceosomal complex). The proximity of its family members to the GO:0070161 family in the UMAP space is illustrated in Fig. 3.D. In the Jaccard layer, these two families are separated by distance. But in the layers of the multiplex network, many of their descendant gene sets (e.g., GO:0014704 and GO:0071006) become variably close to each other. A biological explanation is that spliceosomes actively process pre-mRNA, and the spliced mRNA will impact the product of many molecules, including those related to the anchoring junction. For immune cells, the anchoring junction relates to, for example, cell migration, cell adherence, and antigen presentation. Thus, unsurprisingly, these two families of gene sets interact in context-based networks. Many of their family members aggregate in the embedded UMAP space (Fig. 3.D) in line with their within-family fold change. Thus, the model of multiplex networks of gene sets can provide insights into context-based gene-set relations that significantly differ from their knowledge-based counterpart.

### 3.3 Structural coefficients can provide multifaceted information for gene sets in a multiplex network

**Fig. 4:**
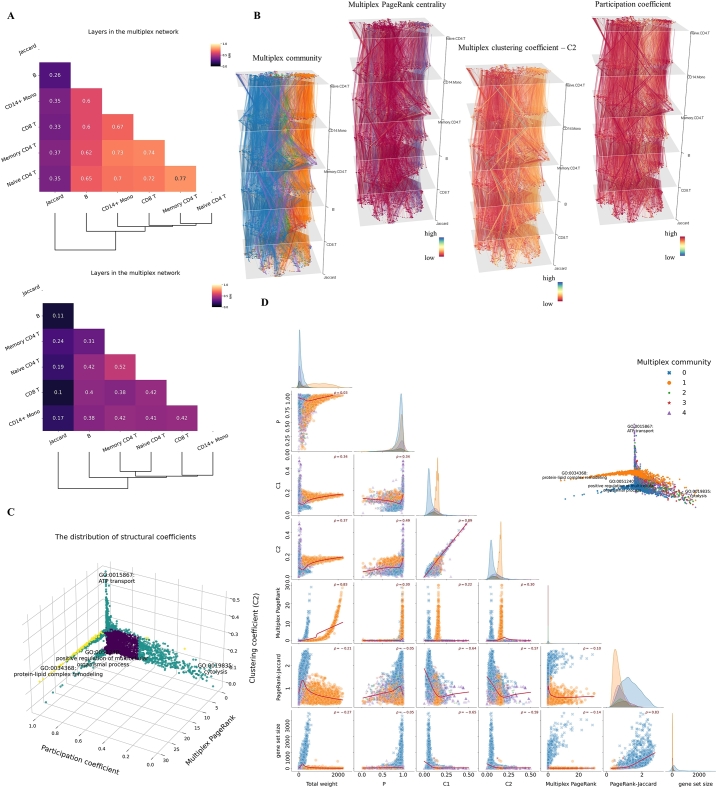
Structural properties of a multiplex network of GO-BP gene sets. (A) NMI scores indicate layer-wise similarities. The dendrogram shows the hierarchical clustering of layers in the multiplex network. Upper: The threshold is 0.2 for filtering edges. Lower: The threshold is relaxed to 0.1 to preserve variations of inter-gene-set relations. (B) Visualizations of the multiplex communities and distributional patterns of structural coefficients. The coordinates of gene sets in each layer are UMAP coordinates adjusted by Grimon. Each layer corresponds to a cell population in the PBMC3K scRNAseq dataset, where the inter-gene-set similarities are measured. (C) The distribution of three primary structural coefficients of GO-BP gene sets. The ratios of centrality coefficients to the uniform mass ($\frac{1}{\text{number of gene sets}}$) rather than raw coefficients are shown. Gene sets with higher than 90th percentile C2 and multiplex PageRank coefficients or those with lower than 10th percentile participation coefficients are marked by dark cyan; gene sets in more than one category are marked by yellow. (D) Even though the Spearman correlations among attributes of gene sets may be high, the relations between structural coefficients are generally complex and non-monotonous, as indicated by the locally weighted linear regression lines (red).

Now, we turn to another study on the multiplex network of GO-BP gene sets. The scRNA-seq dataset for computing $S_{\text{KNN}}$ is pbmc3k.final, which can be easily fetched by SeuratData. Cell populations for measurements include naive CD4 T cells, memory CD4 T cells, CD8 T cells, B cells, and CD14 monocytes (section 2.2). The threshold for truncating edge weights is relaxed to 0.1 to preserve more network variations, which results in low pairwise NMI scores among the layers in the multiplex network. The benefit is to reduce isolated nodes and include more gene sets in the measurements of their structural properties. Nevertheless, the clusters of T cell subtypes and the affinity between CD8 T cells and monocytes are observable in the dendrogram (Fig. 4.A). The knowledge-based Jaccard layer again shows the lowest layer-wise similarities to context-based layers in the multiplex network (Fig. 4.A).

Multiplex community detection reveals five major clusters after merging small groups (less than 200 over 5336 filtered gene sets) of gene sets into one extensive collection. The two most significant communities (blue and orange, communities 0 and 1 in Fig. 4.D) are visibly separated (Fig. 4.B, leftmost). Their primary difference is that the gene sets in community 0 are mainly large gene sets with weaker connections to other gene sets. In comparison, the gene sets in community 1 are usually small gene sets with stronger relations to others (Fig. 4.D, bottom left). Community 4 (purple) is an extensive collection merged from isolated small clusters of gene sets. Interestingly, members in community 4 are mainly small gene sets that possess high multiplex clustering coefficients (Fig. 4.D), indicating they are somehow borderline and tightly interconnected groups in the community. In the auxiliary Jaccard network, the partitions of multiplex communities disappear as they blend, which can be explained by the fundamental difference between a knowledge-based network and a context-based multiplex network. In summary, the multiplex communities in this experiment are primarily determined by their basic structural properties in the network, that is, gene set size and connections.

Several structural coefficients for individual gene sets can be computed, as described in section 2.7. They reflect the diversity of gene sets in their community from a structural perspective. They are concise summaries of gene sets' particular local, global, or dynamic features, as shown in Fig. 4.B (second from the left to the rightmost), which visualizes the patterns of three different structural coefficients in the multiplex network. We provide the code and data in the GitHub repository for these 3D visualizations. GO:0034368 (protein-lipid complex remodeling) is the gene set with the highest multiplex PageRank centrality (ratio of 30.03). The protein-lipid complex has been reported to be a positive regulator of immune cells [57]. GO:0051240 (positive regulation of multicellular organismal process) is the gene set with the highest PageRank centrality (ratio of 2.74) in the Jaccard layer. Compared with GO:0034368, it gives less contextual information, and regardless of which cell population, it has the same PageRank centrality given the same collection of GO-BP gene sets. GO:0019835 (cytolysis) exhibits the lowest value for the participation coefficient, reflecting its biased connections in specific layers. Further investigation shows that the connections between GO:0019835 and other gene sets are significantly more active in the “B cells” layer. GO:0015867 (ATP transport) has the highest level of multiplex clustering coefficients (C2). Its clustering neighbors include several gene sets that are descendants of GO:0006862 (nucleotide transport), for example, GO:0015865 (purine nucleotide transport) and GO:0051503 (adenine nucleotide transport). Rarely, a gene set may occupy high ranks in more than two categories of structural coefficients (yellow in Fig. 4.C).

The association between two structural features of gene sets is generally complicated and non-monotonous (Fig. 4.D). However, we can notice some clear associations. The gene set size significantly influences the PageRank centrality of a gene set in the Jaccard network, as a disproportionate number of gene sets in community 0 (often large gene sets) have higher-than-average PageRank centrality in the Jaccard network, resulting in a Spearman correlation of 0.83 (Fig. 4.D), which is not valid for multiplex PageRank centrality because both small and large gene sets can have high centrality depending on the context (Fig. 4.D). Clustering coefficients C1 and C2 are associated significantly in this experiment. Large gene sets tend to have lower clustering coefficients than do small gene sets. However, their clustering coefficients are unrelated to centrality, as indicated by the two peaks in their co-plots (Fig. 4.C, D). The irrelevance illustrates the fundamental difference between centrality and clustering coefficients, as the former more often represents a dominant role in a hierarchical system. The latter reflects the tightness of inter-connections among members in the neighborhood of a gene set.

### 3.4 Using structural coefficients to reorganize and prioritize enriched terms in GSEA

**Fig. 5:**
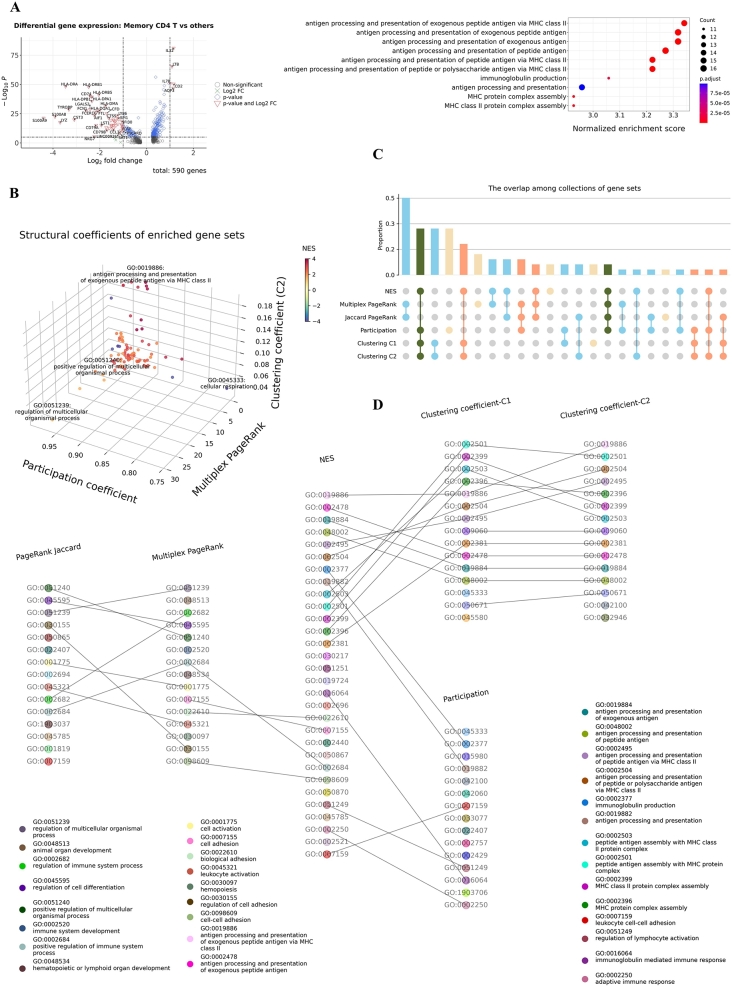
Application of a gene-set multiplex network to GSEA. (A) Left: GSEA experiment on a list of differentially expressed genes. Hundreds of genes are differentially expressed in the memory CD4 T cells (mostly downregulated) compared with other cell populations. Right: Top enriched terms ranked by NES. (B) Distribution of multiplex PageRank centrality, C2 clustering coefficients, and participation coefficients of enriched gene sets. (C) The UpSet plot shows the exclusive intersections among collections of the top 30 enriched gene sets ranked by NES, multiplex/Jaccard PageRank centrality, multiplex clustering coefficients C1 and C2, and participation coefficients. (D) Raw multipartite graph of enriched terms. Many of the top NES enriched gene sets, such as GO:0019886, also have upper-level multiplex clustering coefficients. Some unique gene sets, such as GO:0002684 and GO:0002377, appear at the top of lists by NES and the multiplex PageRank centrality or participation coefficient. However, the raw arrangement of enriched terms has some problems (section 2.8). The first is information redundancy among enriched terms with high clustering coefficients. Second, gene sets with high-level multiplex centrality, such as GO:0002684, are primarily general terms with ambiguous meanings.

The contextual and relational attributes of gene sets obtained by studying a relevant multiplex network can be transferred to GSEA. We run a typical GSEA on a list of differentially expressed genes. The case-control groups are memory CD4 T cells and other cell populations, including naive CD4 T cells, CD8 T cells, B cells, and CD14 monocytes in pbmc3k.final (Fig. 5.A), which are the same groups of cells used for computing similarities and building the multiplex network in section 3.3. The absolute NES values in descending order are used as the criteria to rank enriched GO-BP gene sets (Fig. 5.A). The structural coefficients can be retrieved from the multiplex network built in section 3.3.

The distribution of three structural coefficients of enriched terms in GSEA is displayed in Fig. 5.B. Gene sets with extreme scores in these structural measures, such as GO:0019886, can be easily identified. The UpSet plot visualizes the exclusive intersections among the 6 collections of gene sets that consist of the top 30 gene sets ranked by different standards (Fig. 5.C). A multipartite graph shows the detailed connections between these collections of high-ranking gene sets (Fig. 5.D). High-ranking gene sets identified by the two centrality coefficients or the two clustering coefficients have significant overlapping (Fig. 5.C-D). But for the two centrality coefficients, the multiplex PageRank centrality emphasizes context more; for example, the ranks of immune-related gene sets GO:0002682 and GO:0002684 increase compared to their positions determined by the PageRank centrality in the Jaccard network. Some noticeable gene sets appear in the lists of top NES values and other structural coefficients. For example, GO:0007155 and GO:0022610 (cell/biological adhesion) have upper-level multiplex PageRank coefficients and NES values, and GO:0002377 (immunoglobulin production) has a high NES and a low participation coefficient. Despite these unique findings, some problems are apparent in this raw arrangement of enriched terms. First, many top-ranked items by the NES have redundant information related to antigen processing and presentation (Fig. 5.A, D). Second, the gene sets with top centrality coefficients mainly consist of general terms that need to be more detailed for an in-depth interpretation. For instance, GO:0002684 (positive regulation of the immune system process) relates to the difference between memory T cells and other immune cells. Still, more specific information is needed for interpreting the functional meaning of differentially expressed genes.

**Fig. 6:**
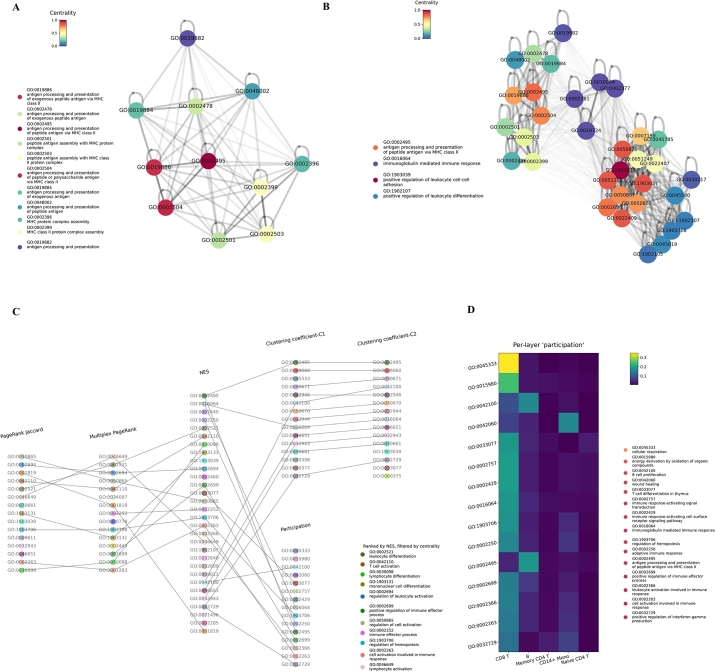
Reorganizing and prioritizing enriched terms by structural coefficients. (A) One group of densely interconnected gene sets. The graph is the embedding of the average adjacency matrix of the multiplex layers. A lighter hue depicts the lines between gene sets with more minor similarities. The color of a node represents its multiplex PageRank centrality in the local network across layers. GO:0002495 exhibits the maximum multiplex centrality among the group members and thus is eligible to represent the group. (B) In the same way, three additional groups are identified, and the local centrality identifies their representative gene sets. The color of a node in this plot represents its multiplex PageRank centrality among all the groups. (C) Organization of enriched terms after compressing groups of enriched terms above and constraining the scope of multiplex centrality to a lower level. Finally, enriched terms are ranked by NES but skipped if they have low-level multiplex centrality. (D) The heatmap visualizes per-layer “participation” of enriched terms with the lowest participation coefficients. A general tendency towards more connectivity in the layer of CD8 T cells is evident.

To solve the first problem, we note that these gene sets also have high-ranking clustering coefficients, which suggests they are located in a densely interconnected region whose members share robust relationships across layers (Fig. 5.D), and a representative term alone may suffice to identify them. In other words, the groups of interconnected gene sets in a multiplex network are likely to share common functions in context and are eligible for being compressed into singular terms. By searching consistent neighbors, the first group of interconnected gene sets are found to include GO:0019886, GO:0002478, GO:0002495, GO:0002501, GO:0002503, GO:0002504, GO:0019884, GO:0019882, GO:0048002, GO:0002396, and GO:0002399. In the GO hierarchical tree, GO:0002501, GO:0002503, GO:0002399, and GO:0002396 are descendant gene sets of GO:0065003 (protein-containing complex assembly), and GO:0019884, GO:0048002, GO:0002504, GO:0002478, and GO:0019886 are descendants of GO:0019882 (antigen processing and presentation). We then compute the (local) multiplex PageRank centrality for members in the group and find that GO:0002495 (antigen processing and presentation of peptide antigen via MHC class II) has the top centrality score. From a biological standpoint, the MHC class II mediates the antigen presentation for immune cells. Thus, GO:0002495 is a reasonable representative for this gene-set group in the multiplex network (Fig. 6.A). Then, the members in the group, except the representative gene set, are removed from the enriched terms. Similarly, we find another three clusters of gene sets, though not exhaustively. Each group of gene sets is represented by a particular gene set with the highest local centrality score (Fig. 6.B). Regarding the second problem, we can adjust the window of centrality to analyze enriched terms with lower multiplex centrality. Thus, we skip the top 15 items in the list of multiplex PageRank centrality, followed by many items with more specific information. The priority of enriched terms is assigned to gene sets that appear on the ranking lists of both NES and multiplex PageRank centrality, such as GO:0002521 (leukocyte differentiation) and GO:0042110 (T cell activation) (Fig. 6.C), which are biologically more informative than the initial list of top enriched terms. We can gain additional insights into enriched terms by checking the gene sets' per-layer “participation” (section 2.8). For example, there is a general tendency of many gene sets for higher participation in the layer of CD8 T cells, for instance, GO:0045333 (cellular respiration) (Fig. 6.D). GO:0042100 (B cell proliferation) and GO:0002495 have more connectivities in the layer of B cells, which concords with the literature that B cells are classical antigen-presenting cells [58]. GO:0042100 (wound healing) has a layer-specificity to the network of CD14 monocytes, and there is evidence to support the role of monocytes in wound healing [59]. These examples suggest that a gene set may connect more with other gene sets in the cell populations where it functions more actively.

In summary, we propose a heuristic pipeline based on the relational attributes of gene sets to reorganize and rank gene sets (section 2.8):

1. Gene sets with high multiplex clustering coefficients (C1 and C2) are clues to searching densely interconnected groups of gene sets. Terms with maximum (local) multiplex centrality can represent other group members so that each group can be compressed into a singular item.
2. Because gene sets with high multiplex PageRank centrality are often general terms with ambiguous meanings (i.e., difficult to interpret), we can adjust the scope to focus on enriched terms with lower multiplex centrality. However, centrality is a sensible measure for prioritizing gene sets. Thus, if an enriched term appears at the top of the NES rank list but has a low multiplex centrality, we can temporarily skip it to find terms with greater centrality.
3. The participation coefficients can assist in a more comprehensive study of enriched terms, as they reflect the biased connectivity of gene sets in different contexts.

Nevertheless, the pipeline is arbitrary, and it may also make sense to follow the order of multiplex centrality or participation coefficients regardless of the NES values. Moreover, the knowledge-based structural coefficients can sometimes approximate the context-based multiplex network model results. But as we demonstrated in section 3.2, the multiplex network model can capture the biological variation of inter-gene-set relations that the knowledge-based model cannot.

## 4 Conclusion

In this study, we explored the new application of a classical KNN-based method to computing the similarity of gene sets. The simulation and running time comparison experiments demonstrated its superior computing performance compared to other similarity measures. Facilitated by $S_{\text{KNN}}$, we built a multiplex network of gene sets to capture biological variation, which better reflects the dynamic and complex nature of gene-set interactions. The structural coefficients of gene sets extract relational characteristics of gene sets in their community, enabling a more thorough understanding of their roles. When researchers perform GSEA, they often have to make a decision on the criteria to prioritize enriched terms to identify the most important ones. Currently, the criteria are monotonous because only the effect size/significance of enrichment is emphasized and gene sets' contextual and relational features are often ignored. However, we can take advantage of these structural coefficients to identify a group of comprehensive criteria to reorganize and prioritize enriched terms, which may enhance the interpretability of GSEA.

## Funding

This work was funded by a KAKENHI Grant-in-Aid from the 10.13039/501100001691Japan Society for the Promotion of Science (Grant No. 21K21316).

## Code availability

The code used in this study is available at https://github.com/flyeous/gene-set-multiplex-network.

## CRediT authorship contribution statement

**Cheng Zheng:** Conceptualization, Formal analysis, Methodology, Software, Validation, Writing – original draft, Writing – review & editing. **Man Wang:** Conceptualization, Methodology. **Ryo Yamada:** Conceptualization, Methodology. **Daigo Okada:** Conceptualization, Methodology, Writing – review & editing.

## Declaration of Competing Interest

The authors declare that they have no known competing financial interests or personal relationships that could have appeared to influence the work reported in this paper.

## Appendix A Proof of equations in $C_{i,1},C_{i,2}$

Recall that $A^{\left[\alpha \right]},A^{\left[\alpha^{\prime }\right]},A^{\left[\alpha^{\prime \prime }\right]}$ are adjacency matrices for three different weighted and directed layers ($G_{\alpha },G_{\alpha^{\prime }},G_{\alpha^{\prime \prime }}$, respectively) in a multiplex network $M=(\text{Y},\overset{\to }{G},G)$ with non-negative, normalized edge weights, and no self-loops. We can write $A^{\prime \;[\alpha ]}=A^{\left[\alpha \right]}+A^{{\left[\alpha \right]}^{T}}$ s.t. $A_{ij}^{\prime \;[\alpha ]}=A_{ij}^{\left[\alpha \right]}+A_{ji}^{\left[\alpha \right]}$ and $A^{\prime \;[\alpha ]}=A^{\prime \;{\left[\alpha \right]}^{T}}$. We treat $A^{\prime \;[\alpha^{\prime }]}$ and $A^{\prime \;[\alpha^{\prime \prime }]}$ similarly in $C_{i,1},C_{i,2}$.

For the numerator of $C_{i,1}$, it suffices to show $\forall \alpha ,\alpha^{\prime }\in Y\;\text{and}\;\alpha \neq \alpha^{\prime }$

$$\sum_{j\neq i,m\neq i,j<m}A_{ij}^{\prime \;[\alpha ]}A_{jm}^{\prime \;[\alpha^{\prime }]}A_{mi}^{\prime \;[\alpha ]}=\frac{1}{2}\sum_{j\neq i,m\neq i,j\neq m}A_{ij}^{\prime \;[\alpha ]}A_{jm}^{\prime \;[\alpha^{\prime }]}A_{mi}^{\prime \;[\alpha ]}\;(\text{by the symmetry of}\;A^{\prime \;[\alpha ]},A^{\prime \;[\alpha^{\prime }]})=\frac{1}{2}\sum_{m\neq i}(\sum_{j\neq m,j\neq i}A_{ij}^{\prime \;[\alpha ]}A_{jm}^{\prime \;[\alpha^{\prime }]})A_{mi}^{\prime \;[\alpha ]}\;(\text{arrangement of terms})=\frac{1}{2}\sum_{m\neq i}{\left(A^{\prime \;[\alpha ]}A^{\prime \;[\alpha^{\prime }]}\right)}_{im}A_{mi}^{\prime \;[\alpha ]}\;(A_{ii}^{\prime \;[\alpha ]},A_{mm}^{\prime \;[\alpha^{\prime }]}=0)=\frac{1}{2}{\left(A^{\prime \;[\alpha ]}A^{\prime \;[\alpha^{\prime }]}A^{\prime \;[\alpha ]}\right)}_{ii}=\frac{1}{2}\mathrm{Diag}{\left(A^{\prime \;[\alpha ]}A^{\prime \;[\alpha^{\prime }]}A^{\prime \;[\alpha ]}\right)}_{ii}$$

For the denominator of $C_{i,1}$, it suffices to show ∀α∈Y

$$\sum_{j\neq i,m\neq i,j<m}A_{ij}^{\prime \;[\alpha ]}A_{mi}^{\prime \;[\alpha ]}=\frac{1}{2}\sum_{j\neq i,m\neq i,j\neq m}A_{ij}^{\prime \;[\alpha ]}A_{mi}^{\prime \;[\alpha ]}\;(\text{by the symmetry of}\;A^{\prime \;[\alpha ]})=\frac{1}{2}\sum_{m\neq i}(\sum_{j\neq m,j\neq i}A_{ij}^{\prime \;[\alpha ]})A_{mi}^{\prime \;[\alpha ]}\;(\text{arrangement of terms})=\frac{1}{2}\sum_{m\neq i}({\left(A^{\prime \;[\alpha ]}1\right)}_{i}-A_{im}^{\prime \;[\alpha ]})A_{mi}^{\prime \;[\alpha ]}\;(A_{ii}^{\prime \;[\alpha ]}=0)=\frac{1}{2}({\left(A^{\prime \;[\alpha ]}1\right)}_{i}{\left(1^{T}A^{\prime \;[\alpha ]}\right)}_{i}-\sum_{m\neq i}A_{im}^{\prime \;[\alpha ]}A_{mi}^{\prime \;[\alpha ]})\;(A_{mm}^{\prime \;[\alpha ]}=0)=\frac{1}{2}{\left({\left(A^{\prime \;[\alpha ]}1\right)}^{\circ 2}-A^{\prime \;{\left[\alpha \right]}^{\circ 2}}1\right)}_{i}\;(\text{by the symmetry of}\;A^{\prime \;[\alpha ]})$$

In a similar way, $\sum_{j\neq i,m\neq i,j\neq m}A_{ij}^{\prime \;[\alpha ]}A_{jm}^{\prime \;[\alpha^{\prime \prime }]}A_{mi}^{\prime \;[\alpha^{\prime }]}=\mathrm{Diag}{\left(A^{\prime \;[\alpha ]}A^{\prime \;[\alpha^{\prime \prime }]}A^{\prime \;[\alpha^{\prime }]}\right)}_{ii}$ $\forall \alpha ,\alpha^{\prime },\alpha^{\prime \prime }\in Y\;\text{and}\;\alpha \neq \alpha^{\prime },\alpha^{\prime \prime }\neq \alpha ,\alpha^{\prime }$ in the numerator of $C_{i,2}$. For the denominator of $C_{i,2}$, it suffices to show $\forall \alpha ,\alpha^{\prime }\in Y\;\text{and}\;\alpha \neq \alpha^{\prime }$

$$\sum_{j\neq i,m\neq i,j\neq m}A_{ij}^{\prime \;[\alpha ]}A_{mi}^{\prime \;[\alpha^{\prime }]}=\sum_{m\neq i}(\sum_{j\neq i,j\neq m}A_{ij}^{\prime \;[\alpha ]})A_{mi}^{\prime \;[\alpha^{\prime }]}\;(\text{arrangement of terms})=\sum_{m\neq i}({\left(A^{\prime \;[\alpha ]}1\right)}_{i}-A_{im}^{\prime \;[\alpha ]})A_{mi}^{\prime \;[\alpha^{\prime }]}\;(A_{ii}^{\prime \;[\alpha ]}=0)={\left(A^{\prime \;[\alpha ]}1\right)}_{i}{\left(1^{T}A^{\prime \;[\alpha^{\prime }]}\right)}_{i}-\sum_{m\neq i}A_{im}^{\prime \;[\alpha ]}A_{mi}^{\prime \;[\alpha^{\prime }]}\;(A_{mm}^{\prime \;[\alpha^{\prime }]}=0)={\left((A^{\prime \;[\alpha ]}1)⊙(A^{\prime \;[\alpha^{\prime }]}1)-(A^{\prime \;[\alpha ]}⊙A^{\prime \;[\alpha^{\prime }]})1\right)}_{i}\;(\text{by the symmetry of}\;A^{\prime \;[\alpha^{\prime }]})$$
